# Hydrogen Gas in Cancer Treatment

**DOI:** 10.3389/fonc.2019.00696

**Published:** 2019-08-06

**Authors:** Sai Li, Rongrong Liao, Xiaoyan Sheng, Xiaojun Luo, Xin Zhang, Xiaomin Wen, Jin Zhou, Kang Peng

**Affiliations:** ^1^Department of Pharmacy, Integrated Hospital of Traditional Chinese Medicine, Southern Medical University, Guangzhou, China; ^2^Nursing Department, Integrated Hospital of Traditional Chinese Medicine, Southern Medical University, Guangzhou, China; ^3^The Centre of Preventive Treatment of Disease, Integrated Hospital of Traditional Chinese Medicine, Southern Medical University, Guangzhou, China

**Keywords:** hydrogen gas, ROS, inflammation, combination, anti-cancer

## Abstract

Gas signaling molecules (GSMs), composed of oxygen, carbon monoxide, nitric oxide, hydrogen sulfide, etc., play critical roles in regulating signal transduction and cellular homeostasis. Interestingly, through various administrations, these molecules also exhibit potential in cancer treatment. Recently, hydrogen gas (formula: H_2_) emerges as another GSM which possesses multiple bioactivities, including anti-inflammation, anti-reactive oxygen species, and anti-cancer. Growing evidence has shown that hydrogen gas can either alleviate the side effects caused by conventional chemotherapeutics, or suppress the growth of cancer cells and xenograft tumor, suggesting its broad potent application in clinical therapy. In the current review, we summarize these studies and discuss the underlying mechanisms. The application of hydrogen gas in cancer treatment is still in its nascent stage, further mechanistic study and the development of portable instruments are warranted.

## Introduction

Gaseous signaling molecules (GSMs) refer to a group of gaseous molecules, such as oxygen (1), nitric oxide (2), carbon monoxide (3), hydrogen sulfide (4), sulfur dioxide (5, 6), ethylene (7, 8), etc. These gaseous molecules possess multiple critical functions in regulating cell biology *in vivo* via signal transduction (9). More importantly, certain GSMs could serve as therapeutic agents in primary cancer, as well as in multidrug-resistant cancer treatment when used by directly or certain pharmaceutical formulations (9–13). In addition, some of these GSMs can be generated in body via different bacteria or enzymes, such as nitric oxide, hydrogen sulfide, indicating that they are more compatible molecules that may exhibit less adverse effects compared with conventional chemotherapeutics (9, 14, 15). Recently, hydrogen gas has been recognized as another important GSM in biology, exhibiting appealing potential in health care for its role in preventing cell injury from various attacking (16–19).

With the formula of H_2_, hydrogen gas is the lightest molecule in the nature which only accounts for about 0.5 parts per million (ppm) of all the gas. Naturally, hydrogen gas is a colorless, odorless, tasteless, non-toxic, highly combustible gas which may form explosive mixtures with air in concentrations from 4 to 74% that can be triggered by spark, heat, or sunlight. Hydrogen gas can be generated in small amount by hydrogenase of certain members of the human gastrointestinal tract microbiota from unabsorbed carbohydrates in the intestine through degradation and metabolism (20, 21), which then is partially diffused into blood flow and released and detected in exhaled breath (20), indicating its potential to serve as a biomarker.

As the lightest molecule in natural, hydrogen gas exhibits appealing penetration property, as it can rapidly diffuse through cell membranes (22, 23). Study in animal model showed that, after orally administration of hydrogen super-rich water (HSRW) and intra-peritoneal administration of hydrogen super-rich saline (HSRS), the hydrogen concentration reached the peak at 5 min; while it took 1 min by intravenous administration of HSRS (23). Another *in vivo* study tested the distribution of hydrogen in brain, liver, kidney, mesentery fat, and thigh muscle in rat upon inhalation of 3% hydrogen gas (24). The concentration order of hydrogen gas, when reached saturated status, was liver, brain, mesentery, muscle, kidney, indicating various distributions among organs in rats. Except the thigh muscle required a longer time to saturate, the other organs need 5–10 min to reach Cmax (maximum hydrogen concentration). Meanwhile, the liver had the highest Cmax (24). The information may direct the future clinical application of hydrogen gas.

Although hydrogen gas was studied as a therapy in a skin squamous carcinoma mouse model back in 1975 (25), its potential in medical application has not been vastly explored until 2007, when Oshawa et al. reported that hydrogen could ameliorate cerebral ischemia-reperfusion injury by selectively reducing cytotoxic reactive oxygen species (ROS), including hydroxyl radical (•OH) and peroxynitrite (ONOO-) (26), which then provoked a worldwide attention. Upon various administrative formulations, hydrogen gas has been served as a therapeutic agent for a variety of illnesses, such as Parkinson's disease (27, 28), rheumatoid arthritis (29), brain injury (30), ischemic reperfusion injury (31, 32), and diabetes (33, 34), etc.

More importantly, hydrogen has been shown to improve clinical end-points and surrogate markers, from metabolic diseases to chronic systemic inflammatory disorders to cancer (17). A clinical study in 2016 showed that inhalation of hydrogen gas was safe in patients with post-cardiac arrest syndrome (35), its further therapeutic application in other diseases became even more appealing.

In the current review, we take a spot on its application in cancer treatment. Typically, hydrogen gas may exert its bio-functions via regulating ROS, inflammation and apoptosis events.

## Hydrogen Gas Selectively Scavenges Hydroxyl Radical and Peroxynitrite, and Regulates Certain Antioxidant Enzymes

By far, many studies have indicated that hydrogen gas doesn't target specific proteins, but regulate several key players in cancer, including ROS, and certain antioxidant enzymes (36).

ROS refers to a series of unstable molecules that contain oxygen, including singlet oxygen (O_2_•), hydrogen peroxide (H_2_O_2_), hydroxyl radical (•OH), superoxide (${•\text{O}}_{2}^{-}$), nitric oxide (NO•), and peroxynitrite (ONOO^−^), etc. (37, 38). Once generated *in vivo*, due to their high reactivity, ROS may attack proteins, DNA/RNA and lipids in cells, eliciting distinct damage that may lead to apoptosis. The presence of ROS can produce cellular stress and damage that may produce cell death, via a mechanism known as oxidative stress (39, 40). Normally, under physical condition, cells including cancer cells maintain a balance between generation and elimination of ROS, which is of paramount importance for their survival (41, 42). The over-produced ROS, resulted from imbalance regulatory system or outer chemical attack (including chemotherapy/radiotherapy), may initiate inner apoptosis cascade, causing severely toxic effects (43–45).

Hydrogen gas may act as a ROS modulator. First, as shown in Ohsawa et al.'s study, hydrogen gas could selectively scavenge the most cytotoxic ROS, •OH, as tested in an acute rat model of cerebral ischemia and reperfusion (26). Another study also confirmed that hydrogen gas might reduce the oxygen toxicity resulted from hyperbaric oxygen via effectively reducing •OH (46).

Second, hydrogen may induce the expression of some antioxidant enzymes that can eliminate ROS, and it plays key roles in regulating redox homeostasis of cancer cells (42, 47). Studies have indicated that upon hydrogen gas treatment, the expression of superoxide dismutase (SOD) (48), heme oxyganase-1 (HO-1) (49), as well as nuclear factor erythroid 2-related factor 2 (Nrf2) (50), increased significantly, strengthening its potential in eliminating ROS.

By regulating ROS, hydrogen gas may act as an adjuvant regimen to reduce the adverse effects in cancer treatment while at the same time doesn't abrogate the cytotoxicity of other therapy, such as radiotherapy and chemotherapy (48, 51). Interestingly, due the over-produced ROS in cancer cells (38), the administration of hydrogen gas may lower the ROS level at the beginning, but it provokes much more ROS production as a result of compensation effect, leading to the killing of cancer cells (52).

## Hydrogen Gas Suppresses Inflammatory Cytokines

Inflammatory cytokines are a series of signal molecules that mediate the innate immune response, whose dys-regulation may contribute in many diseases, including cancer (53–55). Typical inflammatory cytokines include interleukins (ILs) excreted by white blood cells, tumor necrosis factors (TNFs) excreted by macrophages, both of which have shown close linkage to cancer initiation and progression (56–59), and both of ILs and TNFs can be suppressed by hydrogen gas (60, 61).

Inflammation induced by chemotherapy in cancer patients not only causes severely adverse effects (62, 63), but also leads to cancer metastasis, and treatment failure (64, 65). By regulating inflammation, hydrogen gas can prevent tumor formation, progression, as well as reduce the side effects caused by chemotherapy/radiotherapy (66).

## Hydrogen Gas Inhibits/Induces Apoptosis

Apoptosis, also termed as programed cell death, can be triggered by extrinsic or intrinsic signals and executed by different molecular pathways, which serve as one efficient strategy for cancer treatment (67, 68). Generally, apoptosis can be induced by (1) provoking the death receptors of cell surface (such as Fas, TNF receptors, or TNF-related apoptosis-inducing ligand), (2) suppressing the survival signaling (such as epidermal growth factor receptor, mitogen-activated protein kinase, or phosphoinositide 3-kinases), and (3) activating the pro-apoptotic B-cell lymphoma-2 (Bcl-2) family proteins, or down-regulating anti-apoptosis proteins (such as X-linked inhibitor of apoptosis protein, surviving, and the inhibitor of apoptosis) (69, 70).

Hydrogen gas can regulate intracellular apoptosis by impacting the expression of apoptosis-related enzymes. At certain concentration, it can either serve as apoptosis-inhibiting agent via inhibiting the pro-apoptotic B-cell lymphoma-2-associated X protein (Bax), caspase-3, 8, 12, and enhancing the anti-apoptotic B-cell lymphoma-2 (Bcl-2) (71), or as apoptosis-inducing agent via the contrast mechanisms (72), suggesting its potential in protecting normal cells from anti-cancer drugs or in suppressing cancer cells.

## Hydrogen Gas Exhibits Potential in Cancer Treatment

### Hydrogen Gas Relieves the Adverse Effects Related to Chemotherapy/Radiotherapy

Chemotherapy and radiotherapy remain the leading strategies to treat cancer (73, 74). However, cancer patients receiving these treatments often experience fatigue and impaired quality of life (75–77). The skyrocketed generation of ROS during the treatment is believed to contribute in the adverse effects, resulting in remarkable oxidative stress, and inflammation (41, 42, 78). Therefore, benefited from its anti-oxidant and anti-inflammatory and other cell protective properties, hydrogen gas can be adopted as an adjuvant therapeutic regimen to suppress these adverse effects.

Under treatment of epidermal growth factor receptor inhibitor gefitinib, patients with non-small cell lung cancer often suffer with severe acute interstitial pneumonia (79). In a mice model treated with oral administration of gefitinib and intraperitoneal injection of naphthalene which induced severely lung injury due to oxidative stress, hydrogen-rich water treatment significantly reduced the inflammatory cytokines, such as IL-6 and TNFα in the bronchoalveolar lavage fluid, leading to a relieve of lung inflammation. More importantly, hydrogen-rich water didn't impair the overall anti-tumor effects of gefitinib both *in vitro* and *in vivo*, while in contrast, it antagonized the weight loss induced by gefitinib and naphthalene, and enhanced the overall survival rate, suggesting hydrogen gas to be a promising adjuvant agent that has potential to be applied in clinical practice to improve quality of life of cancer patients (80).

Doxorubicin, an anthracycline antibiotic, is an effective anticancer agent in the treatment of various cancers, but its application is limited for the fatal dilated cardiomyopathy and hepatotoxicity (81, 82). One *in vivo* study showed that intraperitoneal injection of hydrogen-rich saline ameliorated the mortality, and cardiac dysfunction caused by doxorubicin. This treatment also attenuated histopathological changes in serum of rats, such as the serum brain natriuretic peptide (BNP), aspartate transaminase (AST), alanine transaminase (ALT), albumin and malondialdehyde (MDA) levels. Mechanistically, hydrogen-rich saline significantly lowered the ROS level, as well as inflammatory cytokines TNF-α, IL-1β, and IL-6 in cardiac and hepatic tissue. Hydrogen-rich saline also induced less expression of apoptotic Bax, cleaved caspase-3, and higher anti-apoptotic Bcl-2, resulting in less apoptosis in both tissues (71). This study suggested that hydrogen-rich saline treatment exerted its protective effects via inhibiting the inflammatory TNF-α/IL-6 pathway, increasing the cleaved C8 expression and Bcl-2/Bax ratio, and attenuating cell apoptosis in both heart and liver tissue (71).

Hydrogen-rich water also showed renal protective effect against cisplatin-induced nephrotoxicity in rats. In the studies, blood oxygenation level-dependent (BOLD) contrast magnetic resonance images (MRI) acquired in different treated group showed that the creatinine and blood urea nitrogen (BUN) levels, two parameters that related to nephrotoxicity, were significantly higher in cisplatin treated group than those in the control group. Hydrogen-rich water treatment could significantly reverse the toxic effects, and it showed much higher transverse relaxation rate by eliminating oxygen radicals (83, 84).

Another study showed that both inhaling hydrogen gas (1% hydrogen in air) and drinking hydrogen-rich water (0.8 mM hydrogen in water) could reverse the mortality, and body-weight loss caused by cisplatin via its anti-oxidant property. Both treatments improved the metamorphosis, accompanied with decreased apoptosis in the kidney, and nephrotoxicity as assessed by serum creatinine and BUN levels. More importantly, hydrogen didn't impair the anti-tumor activity of cisplatin against cancer cell lines *in vitro* and in tumor-bearing mice (85). Similar results were also observed in Meng et al.'s study, as they showed that hydrogen-rich saline could attenuate the follicle-stimulating hormone release, elevate the level of estrogen, improve the development of follicles, and reduce the damage to the ovarian cortex induced by cisplatin. In the study, cisplatin treatment induced higher level of oxidation products, suppressed the antioxidant enzyme activity. The hydrogen-rich saline administration could reverse these toxic effects by reducing MDA and restoring the activity of superoxide dismutase (SOD), catalase (CAT), two important anti-oxidant enzymes. Furthermore, hydrogen-rich saline stimulated the Nrf2 pathway in rats with ovarian damage (86).

The mFOLFOX6 regimen, composed with folinic acid, 5-fluorouracil, and oxaliplatin, is used as first-line treatment for metastatic colorectal cancer, but it also confers toxic effects to liver, leading to bad quality of life of patient (87, 88). A clinical study was conducted in China by investing the protective effect of hydrogen-rich water on hepatic function of colorectal cancer patients (144 patients were enrolled and 136 of them were include in the final analysis) treated with mFOLFOX6 chemotherapy. The results showed that the placebo group exhibited damaging effects caused by mFOLFOX6 chemotherapy as measured by the elevated levels of ALT, AST and indirect bilirubin (IBIL), while the hydrogen-rich water combinational treatment group exhibited no differences in liver function during the treatment, probably due to its antioxidant activity, indicating it a promising protective agent to alleviate the mFOLFOX6-related liver injury (51).

Most of the ionizing radiation-induced adverse effects to normal cells are induced by hydroxyl radicals. The combination of radiotherapy with certain forms of hydrogen gas may be beneficial to alleviate these side effects (89). Indeed, several studies found that hydrogen could protect cells and mice from radiation (48, 90).

As tested in a rat model of skin damage established by using a 44 Gy electronic beam, the treated group by hydrogen-rich water exhibited higher lever of SOD activity and lower MDA and IL-6 in the wounded tissues than the control group and the distilled water group. Furthermore, hydrogen-rich water shortened the healing time and increased the healing rate of skin injury (48).

Gastrointestinal toxicity is a common side effect induced by radiotherapy, which impairs the life quality of cancer patients (91). As shown in Xiao et al.'s study in mice model, hydrogen-water administration via oral gavage increased the survival rate and body weight of mice which were exposed to total abdominal irradiation, accompanied with an improvement in gastrointestinal tract function and the epithelial integrity of the small intestine. Further microarray analysis revealed that hydrogen-water treatment up-regulated miR-1968-5p, which then up-regulated its target myeloid differentiation primary response gene 88 (MyD88, a mediator in immunopathology, and gut microbiota dynamics of certain intestinal diseases involving toll-like receptors 9) expression in the small intestine after total abdominal irradiation (92).

Another study conducted in clinical patients with malignant liver tumors showed that the consumption of hydrogen-rich water for 6 weeks reduced the level of reactive oxygen metabolite, hydroperoxide, and maintained the biologic antioxidant activity in the blood. Importantly, scores of quality of life during radiotherapy were significantly improved in hydrogen-rich water group compared to the placebo water group. Both groups exhibited similar tumor response to radiotherapy, indicating that the consumption of hydrogen-rich water reduced the radiation-induced oxidative stress while at the same time didn't compromise anti-tumor effect of radiotherapy (93).

### Hydrogen Gas Acts Synergistically With Thermal Therapy

Recently, one study found that hydrogen might enhance the effect of photothermal therapy. Zhao et al. designed the hydrogenated Pd nanocrystals (named as PdH_0.2_) as multifunctional hydrogen carrier to allow the tumor-targeted delivery (due to 30 nm cubic Pd nanocrystal) and controlled release of bio-reductive hydrogen (due to the hydrogen incorporated into the lattice of Pd). As shown in this study, hydrogen release could be adjusted by the power and duration of near-infrared (NIR) irradiation. Treatment of PdH_0.2_ nanocrystals plus NIR irradiation lead to higher initial ROS loss in cancer cells, and the subsequent ROS rebound was also much higher than that in normal cells, resulting in more apoptosis, and severely mitochondrial metabolism inhibition in cancer cells but not in normal cells. The combination of PdH_0.2_ nanocrystals with NIR irradiation significantly enhanced the anticancer efficacies of thermal therapy, achieving a synergetic anticancer effect. *In vivo* safety evaluation showed that the injection dose of 10 mg kg^−1^ PdH_0.2_ nanocrystals caused no death, no changes of several blood indicators, and no affected functions of liver and kidney. In 4T1 murine breast cancer tumor model and B16-F10 melanoma tumor model, the combined PdH_0.2_ nanocrystals and NIR irradiation therapy exhibited a synergetic anticancer effect, leading to remarkable tumor inhibition when compared with thermal therapy. Meanwhile, the combination group showed no visible damage to heart, liver, spleen, lung, and kidney, indicating suitable tissue safety and compatibility (52).

### Hydrogen Gas Suppresses Tumor Formation

Li et al. reported that the consumption of hydrogen-rich water alleviated renal injury caused by Ferric nitrilotriacetate (Fe-NTA) in rats, evidenced by decreased levels of serum creatinine and BUN. Hydrogen-rich water suppressed the Fe-NTA-induced oxidative stress by lowering lipid peroxidation, ONOO^−^, and inhibiting the activities of NADPH oxidase and xanthine oxidase, as well as by up-regulating antioxidant catalase, and restoring mitochondrial function in kidneys. Consequently, Fe-NTA-induced inflammatory cytokines, such as NF-κB, IL-6, and monocyte chemoattractant protein-1 were significantly alleviated by hydrogen treatment. More importantly, hydrogen-rich water consumption inhibited several cancer-related proteins expression, including vascular endothelial growth factor (VEGF), signal transducer and activator of transcription 3 (STAT3) phosphorylation, and proliferating cell nuclear antigen (PCNA) in rats, resulting in lower incidence of renal cell carcinoma and the suppression of tumor growth. This work suggested that hydrogen-rich water was a promising regimen to attenuate Fe-NTA-induced renal injury and suppress early tumor events (66).

Non-alcoholic steatohepatitis (NASH) due to oxidative stress induced by various stimuli, is one of the reasons that cause hepatocarcinogenesis (94, 95). In a mouse model, hydrogen-rich water administration lowered the hepatic cholesterol, peroxisome proliferator-activated receptor-α (PPARα) expression, and increased the anti-oxidative effects in the liver when compared with control and pioglitazone treated group (96). Hydrogen-rich water exhibited strong inhibitory effects to inflammatory cytokines TNF-α and IL-6, oxidative stress and apoptosis biomarker. As shown in NASH-related hepatocarcinogenesis model, in the group of hydrogen-rich water treatment, tumor incidence was lower and the tumor volumes were smaller than control and pioglitazone treated group. The above findings indicated that hydrogen-rich water had potential in liver protection and liver cancer treatment (96).

### Hydrogen Gas Suppresses Tumor Growth

Not only working as an adjuvant therapy, hydrogen gas can also suppress tumor and tumor cells growth.

As shown in Wang et al.'s study, on lung cancer cell lines A549 and H1975 cells, hydrogen gas inhibited the cell proliferation, migration, and invasion, and induced remarkable apoptosis as tested by CCK-8, wound healing, transwell assays and flow cytometry. Hydrogen gas arrested the cell cycle at G2/M stage on both cell lines via inhibiting the expression of several cell cycle regulating proteins, including Cyclin D1, CDK4, and CDK6. Chromosomes 3 (SMC3), a complex required for chromosome cohesion during the cell cycle (97), was suppressed by hydrogen gas via ubiquitinating effects. Importantly, *in vivo* study showed that under hydrogen gas treatment, tumor growth was significantly inhibited, as well as the expression of Ki-67, VEGF and SMC3. These data suggested that hydrogen gas could serve as a new method for the treatment of lung cancer (98).

Due to its physicochemical characteristics, the use of hydrogen gas has been strictly limited in hospital and medical facilities and laboratories. Li et al. designed a solidified hydrogen-occluding-silica (H_2_-silica) that could stably release molecular hydrogen into cell culture medium. H_2_-silica could concentration-dependently inhibit the cell viability of human esophageal squamous cell carcinoma (KYSE-70) cells, while it need higher dose to suppress normal human esophageal epithelial cells (HEEpiCs), indicating its selective profile. This effect was further confirmed by apoptosis and cell migration assay in these two cell lines. Mechanistic study revealed that H_2_-silica exerted its anticancer via inducing H_2_O_2_ accumulation, cell cycle arrest, and apoptosis induction mediated by mitochondrial apoptotic pathways (72).

Recently, hydrogen gas was found to inhibit cancer stem cells (CSCs). Hydrogen gas reduced the colony formation and sphere formation of human ovarian cancer cell lines Hs38.T and PA-1 cells via inhibiting the proliferation marker Ki67, stem cell markers CD34, and angiogenesis. Hydrogen gas treatment significantly inhibited the proliferation, invasion, migration of both Hs38.T and PA-1 cells. More important, inhalation of hydrogen gas inhibited the tumor volume significantly as shown in the Hs38.T xenografted BALB/c nude mice model (99).

Another recent study also confirmed the effects of hydrogen gas in suppressing glioblastoma (GBM), the most common malignant brain tumor. *In vitro* study indicated that hydrogen gas inhibited several markers involved in stemness, resulting in the suppression of sphere formation, cell migration, invasion, and colony formation of glioma cells. By inhaling hydrogen gas (67%) 1 h, 2 times per day, the GBM growth was significantly inhibited, and the survival rate was improved in a rat orthotopic glioma model, suggesting that hydrogen might be a promising agent in the treatment of GBM (100).

## Discussion

**Figure 1:**
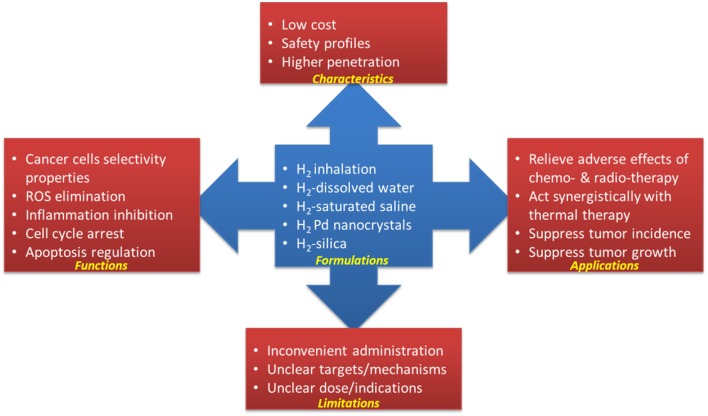
Hydrogen in cancer treatment.

Hydrogen gas has been recognized as one medical gas that has potential in the treatment of cardiovascular disease, inflammatory disease, neurodegenerative disorders, and cancer (17, 60). As a hydroxyl radical and peroxynitrite scavenger, and due to its anti-inflammatory effects, hydrogen gas may work to prevent/relieve the adverse effects caused by chemotherapy and radiotherapy without compromise their anti-cancer potential (as summarized in Table 1 and Figure 1). Hydrogen gas may also work alone or synergistically with other therapy to suppress tumor growth via inducing apoptosis, inhibiting CSCs-related and cell cycle-related factors, etc. (summarized in Table 1).

**Table 1:** The Summary of various formulation, application, mechanisms of H_2_ in cancer treatment.

**Formulation**	**Application**	**Mechanism**	**References**
H_2_-rich water	Prevention of lung injury induced by gefitinib	Inflammatory cytokines and oxidative stress inhibition	(80)
	Prevention of nephrotoxicity induced by cisplatin	Oxygen radicals elimination	(83, 84)
	Reversal of mortality and body-weight loss caused by cisplatin	ROS and apoptosis inhibition	(85)
	Amelioration of liver toxicity induced by mFOLFOX6 regimen	Oxidative stress inhibition	(51)
	Reversal of skin damage established by 44 Gy electronic beam	Inflammatory cytokines and oxidative stress inhibition	(48)
	Amelioration of gastrointestinal toxicity induced by radiotherapy	miR-1968-5p up-regulation	(92)
	Improving the quality of life	Antioxidant activity	(93)
	Renal injury prevention and tumor growth suppression	Inflammatory cytokines and oxidative stress inhibition	(66)
	Tumor incidence and growth suppression	Inflammatory cytokines and oxidative stress inhibition, apoptosis induction	(96)
	Cancer stem cells inhibition	CSCs properties and angiogenesis inhibition	(99)
H_2_-rich saline	Amelioration of cardiac dysfunction induced by doxorubicin	Inflammatory cytokines, ROS and apoptosis inhibition	(71)
	Damage of ovarian cortex induced by cisplatin	Nrf2 pathway stimulation	(86)
H_2_ inhalation	Reversal of toxicity to kidney caused by cisplatin	ROS and apoptosis inhibition	(85)
	Tumor growth suppression	Cell cycle arrest and apoptosis induction	(98)
	Glioblastoma growth inhibition and survival rate enhancement	Inhibition of CSCs properties and induction of glioma stem-like cell (GSC) differentiation	(100)
H_2_ Pd nanocrystals	Synergistic effect with thermal therapy	ROS provoking	(52)
H_2_-silica	Cancer cell viability inhibition	H_2_O_2_ induction, cell cycle arrest, and apoptosis induction	(72)

More importantly, in most of the research, hydrogen gas has demonstrated safety profile and certain selectivity property to cancer cells over normal cells, which is quite pivotal to clinical trials. One clinical trials (NCT03818347) is now undergoing to study the hydrogen gas in cancer rehabilitation in China.

By far, several delivery methods have proved to be available and convenient, including inhalation, drinking hydrogen-dissolved water, injection with hydrogen-saturated saline and taking a hydrogen bath (101). Hydrogen-rich water is non-toxic, inexpensive, easily administered, and can readily diffuse into tissues and cells (102), cross the blood-brain barrier (103), suggesting its potential in the treatment of brain tumor. Further portable devices that are well-designed and safe enough will be needed.

However, regarding its medicinal properties, such as dosage and administration, or possible adverse reactions and use in specific populations, less information is available. Its mechanism, target, indications are also not clear, further study are warranted.

## Author Contributions

SL, XW, JZ, and KP: conceptualization. SL, RL, XS, XL, XZ, JZ, and KP: writing. SL, RL, and XS: revising.

### Conflict of Interest Statement

The authors declare that the research was conducted in the absence of any commercial or financial relationships that could be construed as a potential conflict of interest.
